# Chromosomal and extrachromosomal replicons of a multidrug-resistant zoonotic *Staphylococcus ureilyticus* strain RSM-JSascm isolated from cow milk in Western Uganda, sub-Saharan Africa

**DOI:** 10.1128/mra.00137-25

**Published:** 2025-06-09

**Authors:** Reuben S. Maghembe, Deogratius Mark, Abdalah Makaranga, Samweli Bahati, Jackim Nabona, Maximilian A. K. Magulye, AbdulGaniy. B Agbaje, Claus Thomas, Chinyere Nkemjika Anyanwu

**Affiliations:** 1 Department of Microbiology and Parasitology, Faculty of Medicine, St, Francis University College of Health and Allied Sciences (SFUCHAS) 518753, Ifakara, Tanzania; 2 Omics and Bioinformatics Section, DABA Biotech. Ltd., Dar es Salaam, Tanzania; 3 Department of Microbiology and Immunology, Faculty of Biomedical Sciences, Kampala International University - WC 365672, Ishaka-Bushenyi, Uganda; 4 Tanzania Agricultural Research Institute, Dar-es-Salaam, Tanzania; 5 Phage Team, Kampala, Uganda; 6 Department of Microbiology and Parasitology, Faculty of Medicine, Kairuki University, Dar es Slaam, Tanzania; 7 Department of Biological Sciences, Faculty of Natural and Applied Sciences, Al-Hikmah University 661944, Ilorin, Nigeria; University of Maryland School of Medicine, Baltimore, Maryland, USA

**Keywords:** *Staphylococcus*, genome, chromosome, plasmids, antimicrobial resistance, zoonotic infections, mastitis

## Abstract

*Staphylococcus ureilyticus* is a less-studied pathogen with zoonotic and antimicrobial resistance (AMR) potential. We sequenced a bovine isolate and recovered four replicons, a chromosome with 2,534 coding sequences (CDS), 51 transfer RNAs (tRNA), four ribosomal RNA (rRNA) genes, and two CRISPR arrays, along with three plasmids, harboring AMR genes.

## ANNOUNCEMENT

*Staphylococcus ureilyticus* is a gram-positive coagulase-negative species of the genus *Staphylococcus,* occasionally associated with nosocomial and dairy farm infections (1, 2). Isolates from cow milk have been shown to portray multidrug resistance, encompassing chloramphenicol, erythromycin, fusidic acid, streptomycin, rifampicin, tetracycline, and clindamycin, among others (1, 3). Although *S. aureus* has been implicated in bovine mastitis, the isolation of *S. ureilyticus* from clinics and dairy farms suggests a zoonotic potential. Here, we aimed to recover genomes with virulome and resistome profiles of *Staphylococcus* spp. from dairy samples in Western Uganda.

We sourced raw milk from a vendor 30 min after milking with a machine around Mbarara District (0°36'43.6"S 30°38'14.3"E) and enriched it with brain heart infusion (BHI) broth (HiMedia) for 18 h. This was followed by selective plating on Baird Parker Agar with potassium tellurite and mannitol salt agar and biochemically confirmed as coagulase-negative as described by Deepak and colleagues (3). Genomic DNA was extracted using a Zymo Research Kit as in our previous work (4). A library was constructed on a TruSeq PCR-free kit and sequenced using the Illumina Novaseq X platform to generate a total of 23,158,472 paired-end raw reads (R1 = 1,749,445,532 bp, R2 = 1,739,252,753 bp, each with a 151 bp in length). The reads were quality-controlled with FastQC (v1.1.9) available at https://www.bioinformatics.babraham.ac.uk/projects/fastqc/, assembled with Unicycler v0.4.8 (5) to generate contigs, which were validated with QUAST v5.2.0 (6). The 16S rRNA gene was extracted and used to search for the closest relative with BLASTn (v 2.16.0) (7) against the NCBI nucleotide database (https://blast.ncbi.nlm.nih.gov/Blast.cgi). The closest hit genome was downloaded and then compared with our genome using average nucleotide identity (ANI) with FastANI (8). A chromosome was then reconstructed by scaffolding the contigs of the closest reference genome (accession no. CP094825.1) using MEDUSA v1.0 (9). The chromosome was annotated with RASTk v1.073 (https://rast.nmpdr.org/) and plasmids with PlasmidScope v1.0 (10). Pathogenicity, virulence, and antimicrobial resistance genes were annotated using PathogenFinder (v2.0.4.0) (11), VFanalyzer under the virulence factor gene database (VFDB v2.0, https://www.mgc.ac.cn/cgi-bin/VFs/v5/main.cgi?func=VFanalyzer), and ResFinder (v4.7.2) (12). The replicons were further checked for CRISPR-Cas arrays with CRISPRCasFinder v4.2.2 from CRISPRCasdb (v1.1.2) (13).

**Fig 1:**
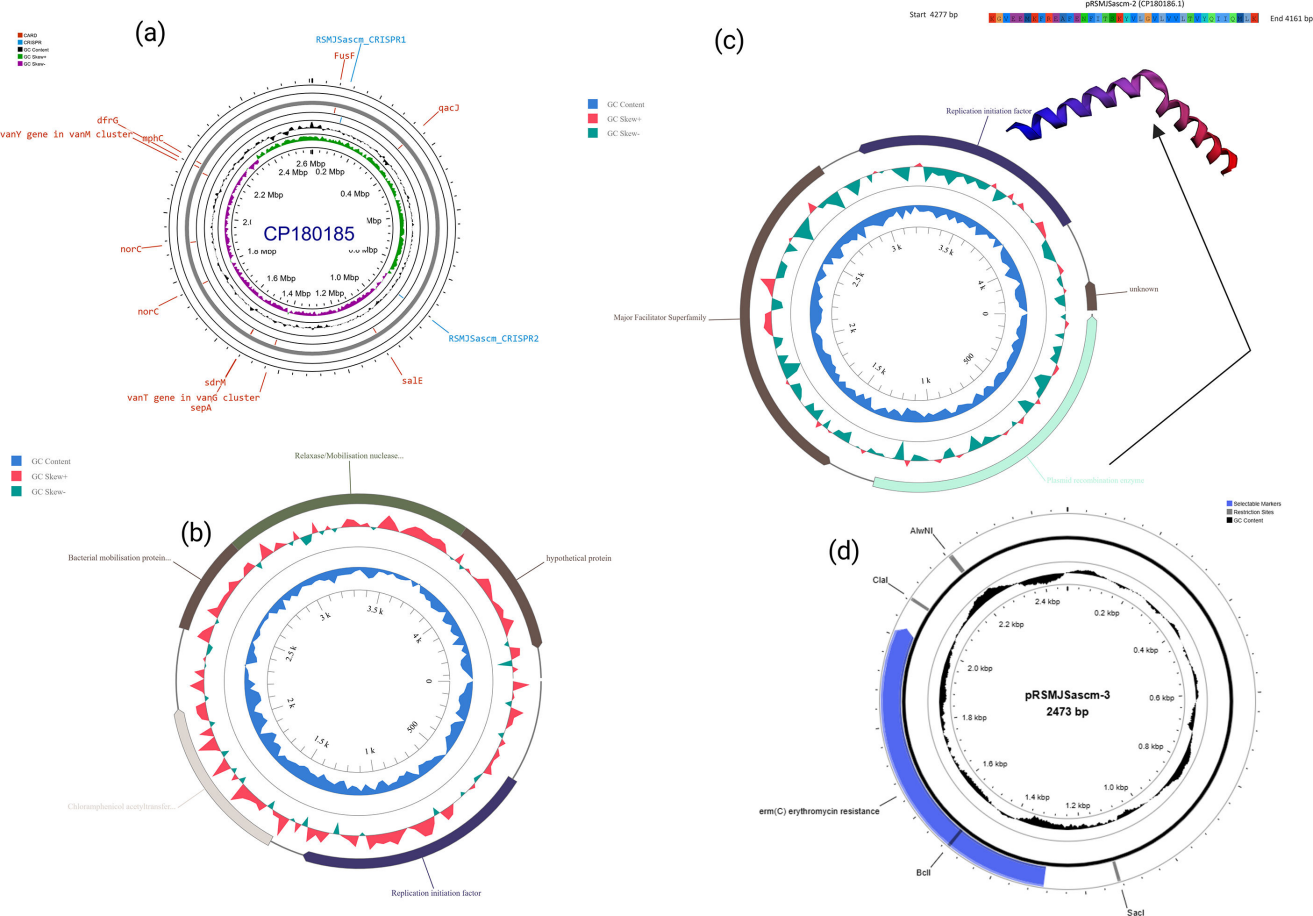
Circular representations of each replicon: (**a**) the chromosome, with AMR and CRISPR-Cas sequence regions indicated, (**b**) plasmid pRSMJScm-1 with all the 4 CDS, (**c**) pRSMJScm-2 with three known proteins, one unknown flanked between 4161 and 4277 bp, and (**d**) the fourth replicon pRSMJScm-3 showing the erm(C) macrolide resistance gene.

Our assembly generated a total of 11 contigs (Table 1), with a GC content of 32.48%, a N50 of 1,045,535, and a completeness of 99.98%. From MEDUSA scaffolding, the contigs were reduced to four replicons. The first replicon, the chromosome (2,608,241 pb), is closely related (ANI = 98.9998%) to the reference *S. ureilyticus* strain IVB6236 (CP094825.1). The results from PathogenFinder suggest that the organism is a potential human pathogen (*P* = 0.977), whose chromosome carries 2,534 coding sequences (CDS) with a virulome of 14 open reading frames (ORFs), 54 RNA genes, two CRISPR-Cas regions, and a phage packaging machinery of 17 genes. In addition, the chromosome harbors *mph(C*) spanning from 2173693 to 2174592 bp, which confers macrolide resistance, and *dfrG (*2160081–2160578 bp), which confers trimethoprim resistance. This chromosome has 2,534 protein-coding sequences (CDS), 51 transfer RNA (tRNA) genes, and four ribosomal RNA (rRNA) genes. Details of annotated features, including those of plasmid replicons, are shown in Fig. 1a through d and summarized in Table 1. The three contigs that remained unmapped to the reference chromosome sequence (CP094825.1) were confirmed to be plasmids through PlasmidFinder v2.1 typing (14), namely pRSMJSascm-1 (4435 bp), pRSMJSascm-2 (4435 bp), and pRSMJSascm-3 (2518 bp). Through BLASTn, all these plasmid replicons matched by 100% with *S. aureus* plasmids rep7b (Accession no. GQ900440), rep7a repC cassette (AB037671), and rep10 repL (pDLK1, accession GU562624), respectively.

**TABLE 1:** Assembly statistics and features of each replicon, including genome size, GC content, annotation of virulence factors, AMR genes, and target antibiotics

Replicon/assembly	No of contigs	Size (bp)	GC (%)	CDS	VF genes	AMR genes	Antibiotics
Total assembly	11	2,619,128	32.48	2546	*atl (orf00497) ebp (orf00909), lip (orf02453), sspA (orf01882), capB (orf00747), capC (orf01823) galE (orf01567), cylR2 (orf02338), vctC (orf00250*)	*mph(C) and dfrG, cat(pC233*), *tet(K), erm(C*)	Trimethoprim, erythromycin, spiramycin, telithromycin, chloramphenicol, tetracycline, clindamycin, and lincomycin
Chromosome		2,608,241	32.48	2,534	*atl (orf00497), ebp (orf00909*), l*ip (orf02453*), *sspA (orf01882*), *capB (orf00747), capC (orf01823) galE (orf01567), cylR2 (orf02338), vctC (orf00250*)	*mph(C) and dfrG*	Trimethoprim, erythromycin, spiramycin, and telithromycin
pRSMJSascm-1		4435	31.25	5	None	*cat(pC233*)	Chloramphenicol
pRSMJSascm-2		4279	29.73	3	None	*tet(K*)	Tetracycline
pRSMJSascm-3		2518	30.77	4	None	*erm(C*)	Clindamycin, erythromycin, and lincomycin

## Data Availability

This Whole Genome Shotgun project has been deposited in DDBJ/ENA/GenBank under the accession no. PRJNA1214672. All the replicons were deposited into GenBank and can be accessed through the accession numbers CP180185 (chromosome), CP180186.1 (pRSMJSascm-1), CP180187 (pRSMJSascm-2) and CP180188 pRSMJSascm-3). The raw reads can be accessed from the Sequence Read Archive (SRA) with accession no. SRX27508106, and BioSample no. SAMN46506376.
